# Exploring Factors Influencing Pregnant Women’s Perceptions and Attitudes Towards Midwifery Care in Romania: Implications for Maternal Health Education Strategies

**DOI:** 10.3390/nursrep14030134

**Published:** 2024-07-23

**Authors:** Mihaela Corina Radu, Mihai Sebastian Armean, Melania Pop-Tudose, Cosmin Medar, Loredana Sabina Cornelia Manolescu

**Affiliations:** 1 Department of Microbiology, Parasitology and Virology, Faculty of Midwives and Nursing, “Carol Davila” University of Medicine and Pharmacy, 020021 Bucharest, Romania; 2 Constantin Andreoiu Emergency Hospital County, 100409 Ploieşti, Romania; 3 Department of Pharmacology, Toxicology and Clinical Pharmacology, “Iuliu Hateganu” University of Medicine and Pharmacy, 400347 Cluj Napoca, Romania; sebastian.armean@umfcluj.ro; 4 Department of Obstetrics and Gynecology, Faculty of Midwives and Nursing, “Carol Davila” University of Medicine and Pharmacy, 020021 Bucharest, Romania; melaniaelenatudose@gmail.com; 5 Department of Fundamental Sciences, Faculty of Midwifery and Nursing, “Carol Davila” University of Medicine and Pharmacy, 050474 Bucharest, Romania; cosmin.medar@umfcd.ro; 6 “Profesor Dr. Th. Burghele” Clinical Hospital, Department of Clinical Laboratory of Radiology and Medical Imaging, 050664 Bucharest, Romania

**Keywords:** midwifery, midwifery care, maternal health, education, public health, prenatal courses

## Abstract

Background: Midwives are strong advocates for vaginal births. However, their visibility and accessibility are poorly perceived by women in Romania. Consequently, the women’s options are limited to a single direction when pregnancy occurs, involving the family doctor, the obstetrician, and often an interventional technical approach at the time of birth. The aim of this research is to identify specific variables that affect the perceptions and attitudes of pregnant women towards the care provided by midwives. This knowledge could contribute to the development of more effective education and information strategies within maternal health services. Methods: A cross-sectional observational analytical survey was conducted in Romania among pregnant women from the general population. Data were collected through a self-administered questionnaire, with informed consent obtained from each participating pregnant woman. The questionnaire was administered online using the cloud-based Google Forms platform and was available on the internet for seven months, from January to July 2023. The questionnaire was distributed through various media channels, both individually and in communication groups, in the form of a link. All questions were mandatory, and the questionnaire could only be submitted after answering all questions. Results: A total of 1301 individual responses were collected. The analysis of the socio-demographic and obstetrical profile of the pregnant women revealed that approximately half, 689 (52.95%), of the participants were aged between 18–29 years, and 1060 (81.47%) of the participants were married. Among our group of 1301 pregnant women, 973 (74.78%) had higher education, and 987 (75.86%) had a regular job. A majority of the survey participants, 936 (71.94%), lived in an urban geographic area, while 476 (36.58%) had attended childbirth education courses, and 791 (60.79%) were in the third trimester of pregnancy. A total of 298 (22.9%) respondents did not want to give birth in a hospital, and one-third, 347 (26.67%), did not place significant importance on control over the childbirth process. Conclusions: The main factors influencing women’s decisions regarding perinatal care and the importance of midwives as a component of the maternal-infant care team are modifiable, and thorough educational and psychological preparation would reduce the increasing predominance of preference for cesarean section, thereby promoting healthier and more woman- and child-centered perinatal care.

## 1. Introduction

Maternity services hold a unique position in influencing the current and future health of mothers and infants, with midwives playing an essential role in this regard.

The well-being of mothers and children lays the foundation for a healthy future. However, maternal and infant mortality during the prenatal and perinatal period, encompassing pregnancy, birth, and miscarriage, remains a concern in Romania [1,2,3,4,5].

To address this issue, the involvement of midwives is crucial. Given the significance of public health, maternity services possess a unique capacity to shape the present and future well-being of mothers and children, with midwives playing a pivotal role. In numerous healthcare systems worldwide, midwives are employed to monitor pregnancies, both from physiological and pathological perspectives, including aspects such as TORCH (T—Toxoplasmosis: Toxoplasmosis O—Other (such as syphilis, varicella-zoster, parvovirus B19, and others): Other (such as syphilis, varicella-zoster, parvovirus B19, and others) R—Rubella: Rubella C—Cytomegalovirus: Cytomegalovirus H—Herpes simplex: Herpes simplex—infections that are often tested together because they can cause serious problems during pregnancy), while also assisting with facilitating vaginal childbirth [6,7,8].

Nonetheless, effective pregnancy monitoring stands as the cornerstone for achieving a complication-free birth.

### An Overview of Maternity Services in Romania

The current population of Romania is 19,618,996 (as of 11 May 2024). In 2022, there were 182,083 recorded births [9,10]. Maternity services in Romania thus play an essential role, with 178,617 births occurring in hospitals, 359 births at home, and 3107 categorized by the National Institute of Statistics as other situations, likely referring to births in private hospitals. Of the over 180,000 births, only 2740 were attended solely by midwives, while 116,012 were attended by both a doctor and a midwife. The number of births in each of the 42 counties of the country ranges from 17,344 in Bucharest to 1454 in Tulcea County, with 88,125 births occurring in urban areas [3].

In Romania, healthcare is regulated by Law 95/2006, the framework health law, and is organized as a mandatory health insurance system [11]. The central authority at the national level is the Ministry of Health, which is responsible for developing and planning health policies, proposing legislation, and overseeing the entire sector, while county-level authorities supervise the organization and provision of services for the population.

Primary maternal and infant care is provided by family doctors (monitoring the progress of pregnancy and postpartum), while specialized outpatient care is provided by obstetricians. The profession of a licensed midwife in Romania has officially existed in the Romanian Occupation Code since 2011, under the number 222,201, and requires a license to be obtained through graduation from the relevant faculties of medical universities. The midwife was included in the community care team by Law 180/2018. Antenatal and postnatal care in Romania, the methodology of consultations during these periods, the conditions for providing primary and specialized care, service packages within the social health insurance system, and medical documents completed by medical personnel during pregnancy, namely the pregnant woman’s booklet and the pregnant woman’s file, are all regulated by law [11,12].

Within the Romanian social health insurance system, pregnant women, regardless of their insurance status, are entitled to ten consultations (initial registration in the first trimester, one consultation per month from the third to the seventh month, and two consultations per month from the seventh to the ninth month) and postpartum women are entitled to two consultations (at discharge from the maternity hospital and 4 weeks after birth) at the primary healthcare level, and one consultation per trimester of pregnancy, and one postnatal consultation at the specialized outpatient level (obstetrician), reimbursed by the National Health Insurance Fund. These consultations are performed by the family doctor and the obstetrics–gynecology specialist, as well as by other specialists depending on the conditions presented by the pregnant woman, with midwives not included in the care team [13].

According to Romanian legislation, childbirth—regardless of the type of delivery—is covered by the mandatory social health insurance system for both insured and uninsured women (basic and minimum health insurance packages). Therefore, a woman and her family will not incur any expenses related to childbirth in public hospitals. Some public hospitals may offer patients the option, strictly voluntarily, to benefit from special accommodation conditions (private room). In these situations, the patient will directly pay the accommodation cost [13].

Generally, in the public maternity system, a woman is normally assisted by an obstetrician. If the obstetrician determines that the birth is considered low risk, meaning it is an uncomplicated birth, it may be assisted by a midwife under the obstetrician’s supervision [10].

Women who choose to give birth in a private facility will need to pay a price that depends on the type of delivery and the clinic’s commercial policy. The price for a caesarean section is higher than for a vaginal birth, and fees vary from clinic to clinic. Some private hospitals have contracts with the National Health Insurance House. Birth services are reimbursed at the level of public hospitals, but there are usually additional fees for clients without private health insurance [1,4,10,14,15,16].

Private maternity care is typically accessed only by women who have private health insurance or can afford to pay for medical services. In the private sector, a woman usually receives prenatal care through obstetric practices of specialist doctors who may also work in state hospitals [10,17].

Women have the greatest trust in their obstetrician. The obstetrician monitoring the pregnancy is the primary educated source of information and the key factor influencing women’s decisions regarding the type of birth; family doctors and midwives play a minimal role in the decision-making process. Typically, women choose an obstetrician to monitor the progress of their pregnancy and follow this doctor into private settings for prenatal consultations if they can afford it. Most obstetricians working in public hospitals also offer consultations in private clinics. Sometimes, doctors in the public system refer women to their private practices. Often, women pay directly for prenatal Doppler ultrasounds and other tests and consultations during pregnancy that cannot be provided by public health insurance [1,4,10,14,15,18].

In Romania, prenatal courses exist, but nothing is standardized. In different maternity wards, there are midwives or clinicians involved in such activities. The topics are chosen at their discretion, based on their own experience. Prenatal education about childbirth is mostly paid for directly by interested individuals and is mainly organized by hospitals and private clinics [19].

According to Article 42 of the European Directive 36/2005/EC, amended by DE 55/2013 and identically transposed into Romanian legislation, midwives are authorized to provide specific maternal and infant care, including the full range of antenatal and postnatal services for healthy pregnant women, postpartum women, and newborns [20].

According to the National Institute of Statistics, in 2022, there were 2121 midwives and Obstetrics–Gynaecology nurses (with the last certification in 1994) only in specialized institutions, and no midwives in antenatal and postnatal care. Additionally, there were 7000 obstetricians, and family medicine accounted for 12,000 (60% urban, 40% rural—6700 offices compared to 4600 offices in rural areas) for 180,000 births [3,21].

The number of midwives is insufficient for several reasons. The profession requires a better regulatory framework, policies, and measures that enable them to practice at all levels of care including primary healthcare, outpatient, and hospital settings. The current regulatory framework lacks clarity in many aspects, ranging from malpractice legislation to methodologies and tools used to evaluate the effectiveness, accessibility, and cost of prenatal care. There is also a need for interventions to improve women’s medical education and knowledge [22].

Midwives are strong advocates for vaginal births [23]. Women would have more confidence in midwives if they were available in both community and hospital settings, offering education and prenatal care. There is a multitude of recent and high-quality evidence demonstrating the impact and effectiveness of midwives in enhancing sexual and reproductive health outcomes. The 2014 State of the World’s Midwifery report concluded that midwives, when educated and regulated to international standards, can address 87% of the global need for sexual and reproductive health services [24].

However, their visibility and accessibility are poorly perceived by women in Romania. Consequently, the women’s options are limited to a single direction when pregnancy occurs, involving the family doctor, the obstetrician, and often an interventional technical approach at the time of birth.

The aim of this article is to identify specific variables that affect the perceptions and attitudes of pregnant women towards care provided by midwives. This knowledge could contribute to the development of more effective education and information strategies within maternal health services.

## 2. Materials and Methods

A cross-sectional observational analytical survey was conducted in Romania among pregnant women from the general population. To ensure a rigorous design, we followed the guidelines of “Strengthening the Reporting of Observational Studies in Epidemiology” (STROBE).

### 2.1. Inclusion Criteria

This study targeted pregnant women from the general population in Romania, residing in the counties and the capital of the country. The inclusion criteria for pregnant participants were a minimum age of 18 years, Romanian citizenship, no severe chronic conditions, and no history of infertility. We excluded 18 participants because they were under 18 years of age.

### 2.2. Survey Questionnaire and Data Collection

Data were collected through a self-administered questionnaire, with informed consent obtained from each participating pregnant woman. The questionnaire was administered online using the cloud-based Google Forms platform and was available on the internet for seven months, from January to July 2023. The questionnaire was distributed through various media channels, both individually and in communication groups, in the form of a link. After completion in Google Forms, informed consent was requested and obtained from each participant willing to complete the questionnaire, initially informing them about the survey’s purpose. All questions were mandatory, and the questionnaire could only be submitted after answering all questions.

A total score was calculated for the following questions:

1. Are you aware of the services that midwives can provide during pregnancy, childbirth, and postpartum? Possible scores ranged from 0 (complete lack of information) to 2 (sufficient information).
2. In your opinion, would access to a midwife during pregnancy, childbirth, and postpartum be helpful? Possible scores identical to the scores for the previous question.
3. On a scale from 1 to 10 (where 1 is the least important and 10 is very important), how important do you consider the midwife to be in the stages of pregnancy, childbirth, and postpartum? The score was based on the scale.

The total possible score ranged from 1 (the patient is not informed at all about the role of the midwife during pregnancy/labor–delivery/postpartum or considers the role to be unimportant) to 14 (the patient is fully aware of the midwife’s role during pregnancy/labor–delivery/postpartum). The total score obtained was compared for subsamples of patients determined by their demographic and clinical characteristics.

### 2.3. Measures of Demographic Characteristics

Women were asked for information about their age, ethnicity, residence, income, current marital status, and highest educational qualification achieved.

### 2.4. Measure of Health Status

Women were asked to indicate if they were currently pregnant and, if so, at what stage of pregnancy (i.e., trimester), with the absence of pregnancy being an exclusion criterion. Women were also asked to indicate if they had previously had a viable birth and the type of delivery (i.e., vaginal birth, cesarean section).

Women were asked to indicate the frequency of consultations during the current pregnancy with a family doctor and/or a specialist doctor from the public or private sector. Additionally, they were asked to specify their preferred place of birth and their expectations regarding autonomy in decisions during pregnancy and childbirth.

### 2.5. Statistical Analysis

For statistical analysis, JASP 0.18.3 and R © JASP Team (2024) were used. JASP (Version 0.18.3) [Computer software] and R version 4.3.3 Copyright (C) 2024, The R Foundation for Statistical Computing, R Core Team (2024). R: A language and environment for statistical computing. R Foundation for Statistical Computing, Vienna, Austria, with the following packages: EFA.dimensions [25], EFAtools [26], lavaan [27], semPlots [28], and gtsummary [29].

For data processing, the COUNTIFS function in Microsoft Office Excel was used to filter and sort the initial database. Categorical variables related to the socio-demographic characteristics of pregnant women and their obstetric profile were expressed using descriptive statistics and frequencies. Bidirectional Welch T-tests were used if the categorical variable had two categories, or ANOVA tests followed by post hoc procedures if the categorical variable had more than two categories. A *p*-value < 0.05 was adopted for statistical significance.

### 2.6. Ethical Approval

The questionnaire was peer-reviewed and approved by the Ethics Committee of the Dr. Constantin Andreoiu Emergency Hospital in Ploiești, Romania (41482/09.08.2022); all study procedures adhered to the ethical standards of the Declaration of Helsinki. Informed consent was mandatory.

## 3. Results

A total of 1301 individual responses were collected. The responses to the questions are summarized in the Supplementary Materials File.

The analysis of the socio-demographic and obstetrical profile of the pregnant women revealed that approximately half, 689 (52.95%), of the participants were aged between 18 and 29 years, and 1060 (81.47%) of the participants were married. Among our group of 1301 pregnant women, 973 (74.78%) had higher education, and 987 (75.86%) had a regular job. A majority of the survey participants, 936 (71.94%), lived in an urban geographic area, while 476 (36.58%) had attended childbirth education courses, and 791 (60.79%) were in the third trimester of pregnancy. A total of 298 (22.9%) respondents did not want to give birth in a hospital, and one-third, 347 (26.67%), did not place significant importance on control over the childbirth process (Table 1 and Table 2).

## 4. Discussion

This study is the first in Romania to analyze the perceptions and attitudes of pregnant women regarding the importance of midwives during pregnancy, childbirth, and the postpartum period. Our study results indicate that women who place greater importance on midwives in perinatal care are over 40 years old. According to recent studies, there is a correlation between the increasing average age of pregnant women and the rising number of cesarean interventions, and this aspect can be associated with concerns about risks, as older women are more likely to experience pregnancy complications [30,31]. However, the experience they have accumulated enables them to better evaluate the risks and benefits of different care options, leading to more informed and suitable choices for their individual needs. Some studies suggest that some women have reported that receiving additional information about age-related risks is unnecessary and causes anxiety because their age is an unmodifiable risk factor [32,33,34,35]. Other studies suggest that women aged 35 and older may desire more participation and control over their care than younger women and are more capable of achieving this involvement [36,37].

Older women who have had multiple pregnancies and births have accumulated direct experience with the physiological processes involved in pregnancy and childbirth. This experience provides them with a practical understanding of bodily changes and the specific needs of each stage of pregnancy, which can positively influence their perception of the care provided by midwives, as they are already familiar with the childbirth process and the role of midwives in it. These findings highlight the importance of tailoring perinatal care to the individual needs and experiences of each woman.

This study demonstrated a correlation between the number of previous births and satisfaction with perinatal care. Other research has shown that women with previous births are often more satisfied with the care received from midwives due to their prior experience and well-calibrated expectations. Studies indicate that first-time mothers particularly benefit from the care provided by midwives, due to the intense support and continuous education offered, which significantly enhances their childbirth experience and increases their satisfaction [38,39,40].

The study highlighted the fact that married women have a positive attitude towards the involvement of midwives in perinatal care. These results are supported by other studies that have analyzed the influence of marital status on the preferences and perceptions related to perinatal care [41,42,43].

Married women or those in stable relationships may benefit from more consistent support from their partner, which can positively influence their attitude towards midwives’ involvement. This emotional and practical support can reduce anxiety related to pregnancy and childbirth, facilitating the acceptance of a more natural and personalized approach offered by midwives. In contrast, single women or those without a stable partner may face additional challenges that affect their quality of life and overall well-being during pregnancy. The absence of a stable partner can amplify feelings of vulnerability and insecurity, leading to a greater need for extended professional support. In line with women’s expectations, other studies on the impact of male involvement on maternal health outcomes have found that male involvement was significantly associated with reduced chances of postpartum depression and improved utilization of skilled birth attendance and postnatal care [44,45].

Additionally, satisfaction with the care provided by midwives among women who participated in childbirth education programs, compared to those who did not participate, shows statistically significant differences in our study. In our study, there is a significant importance placed on midwives by women who participated in childbirth education courses. These findings underscore the importance of education in promoting quality perinatal care and adequate support for all women and their partners, regardless of their level of education. Numerous studies have shown that education plays a crucial role in shaping the perceptions and preferences related to perinatal care, highlighting the need to promote health education and facilitate access to accurate and up-to-date information for all women. Research has shown that women who participate in childbirth education programs report a more positive birth experience and greater satisfaction with the role of midwives [46,47,48,49].

Both the current research and other studies have confirmed that the level of education significantly influences attitudes towards perinatal care. Women with higher education are more likely to opt for midwifery care due to awareness of the associated benefits [50,51,52]. Midwives can provide detailed and personalized information tailored to patients’ level of understanding, as educated patients are better able to voice their concerns, seek clarification, and make informed decisions about their care. This can lead to a trusting relationship and better collaboration in care. Maternal education also varies by age. Although another study found no association between education and perceptions of midwives’ involvement in maternal care, mothers with lower levels of education tended to be more critical and less satisfied with the quality of maternal care and higher levels of education among the older mothers may enable them to achieve more effective and satisfying communication than younger women [53].

Additionally, women with university education are more likely to feel responsible for their own health, possibly due to increased education, leading to a greater interest in their health status [54,55,56,57,58].

Higher education develops critical thinking and decision-making skills, allowing women to better evaluate perinatal care options and make informed choices. These women are better able to compare different care models and select the one that aligns best with their values and preferences. They can communicate more effectively with healthcare professionals and request personalized care, including involving midwives in the care team [59].

Studies highlighting women’s less medicalized and more positive birth experiences with a midwife or at home, as well as the lower costs of intrapartum care [60,61,62], question the care models in the Romanian healthcare system. In this system, pregnant women only have the option of state or private hospitals for childbirth. Our study, which highlighted the importance placed on midwives and their presence in perinatal care among respondents who prefer a birth location other than public or private hospitals, underscores the need to reassess and diversify the options available to women. Our research results raise significant questions about the level of satisfaction of women with the current model of perinatal care in Romania. According to studies, the main reasons women opt for home births are the intimacy and safety of home, the presence of a companion, and the desire to experience a natural and free birth. These aspects should prompt reflection on how the healthcare system can better respond to women’s needs and preferences during childbirth [63].

The interpretation of our findings may be potentially limited by the fact that data on the importance of midwives in the Romanian healthcare system are self-reported by participants, and as such, our results could be subject to bias effects.

## 5. Conclusions

In conclusion, the main factors influencing women’s decisions regarding perinatal care and the importance of midwives as part of the maternal-infant care team are modifiable, leading to the following recommendations: thorough educational and psychological preparation to reduce the increasing preference for cesarean sections, proper implementation of the midwifery profession and increased accessibility to prenatal care with midwives to reduce reliance on technical interventional approaches, inclusion of accurate information about the benefits, risks, and dangers associated with each type of birth in prenatal care and education, and maternal health programs should include specific initiatives that encourage partner participation. By implementing these recommendations, a larger amount of healthier woman- and child-centered perinatal care options can be promoted.

## Figures and Tables

**Table 1:** Distribution of demographic, clinical characteristics and the importance of the midwife’s role during pregnancy/childbirth/postpartum among respondents.

	Category	Mean (SD)	Welch’s *t*-Test/Anova	*p* Value
Age	18–29 years (N = 689)	10.70 (3.00)	F(1, 1299) = 18.28	*p* < 0.0001
	30–39 years (N = 570)	11.10 (2.96)		
	Over 40 years (N = 42)	12.80 (1.17)		
Place of Residence	Rural (N = 365)	10.80 (2.99)	T(657) = −1.26	*p* = 0.208
	Urban (N = 936)	11.00 (2.95)		
Level of Education	Primary/Middle School (N = 75)	9.53 (4.04)	F(1, 1299) = 0.22	*p* = 0.013
	High School (N = 252)	11.20 (2.72)		
	Higher Education (N = 973)	11.00 (2.90)		
Occupational Status	Stable Occupation (N = 987)	11.00 (2.89)	T(488) = 1.53	*p* = 0.127
	Occasional/No Occupation (N = 314)	10.70 (3.18)		
Ethnicity	Romanian (N = 1260)	10.90 (2.99)	T(46.50) = 0.23	*p* = 0.814
	Other Ethnicity (N = 41)	11.00 (1.92)		
Marital Status	Married (N = 1060)	11.00 (2.89)	T(331) = −2.25	*p* = 0.025
	Unmarried/Widowed/ Divorced (N = 241)	10.50 (3.26)		
Previous Births	No previous births (N = 800)	10.60 (2.92)	F(1, 1299) = 23.20	*p* < 0.0001
	1–2 previous births (N = 464)	11.40 (2.94)		
	More than 3 previous births (N = 37)	11.50 (3.31)		
Participation in Educational Programs	Yes (N = 476)	11.70 (2.84)	T(1019) = 7.79	*p* < 0.0001
	No (N = 825)	10.50 (2.94)		
Place of Childbirth	Public Hospital (N = 596)	10.975 (3.23)	F(1, 1299) = 10.476	*p* < 0.001
	Private Hospital (N = 407)	12.092 (2.45)		
	Other Location (N = 298)	13.200 (0.76)		
Importance of Control Over the Birth Process	Very Important (N = 605)	12.143 (2.13)	F(1, 1299) = 41.475	*p* < 0.001
	Somewhat Important (N = 349)	9.932 (3.44)		
	Not Important (N = 347)	9.275 (4.41)		
Trimester of Pregnancy	First Trimester (N = 176)	11.30 (2.45)	F(1, 1299) = 2.96	*p* = 0.086
	Second Trimester (N = 340)	10.10 (3.01)		
	Third Trimester (N = 791)	11.20 (2.98)		

**Table 2:** Distribution of respondents according to the importance they attach to the presence of the midwife during pregnancy, childbirth, and the postpartum period.

Question	Answer	% N
Are you aware of the services that midwives can provide during pregnancy, childbirth, and postpartum?	Yes, I am well informed about their services	56.87% N = 740
	I have some knowledge about their services	6.91% N = 90
	No, I don’t know their services	36.20% N = 471
In your opinion, would access to a midwife during pregnancy, childbirth, and postpartum be helpful?	Yes	81.16% N = 1056
	No	3.68% N = 48
	I do not know	15.14% N = 197
On a scale from 1 to 10 (where 1 is the least important and 10 is very important), how important do you consider the midwife to be in the stages of pregnancy, childbirth, and postpartum?	Is the important 1	2.15% N = 28
	Is the important 2	0.30% N = 4
	Is the important 3	2.15% N = 28
	Is the important 4	1.22% N = 16
	Is the important 5	8.30% N = 108
	Is the important 6	2.76% N = 36
	Is the important 7	6.84% N = 89
	Is the important 8	18.52% N = 241
	Is the important 9	9.76% N = 127
	Is the important 10	47.96% N = 624

## Data Availability

The raw data supporting the conclusions of this article will be made available by the authors on request.

## Supplementary Materials

The following supporting information can be downloaded at: https://www.mdpi.com/article/10.3390/nursrep14030134/s1.

## Public Involvement Statement

No public involvement in any aspect of this research.

## Guidelines and Standards Statement

A complete list of reporting guidelines can be accessed via the equator network: https://www.equator-network.org/ (accessed on 1 June 2024).
